# Costimulatory T cell engagement via a novel bispecific anti-CD137 /anti-HER2 protein

**DOI:** 10.1186/2051-1426-3-S2-P187

**Published:** 2015-11-04

**Authors:** Marlon J Hinner, Rachida-Siham Bel Aiba, Alexander Wiedenmann, Corinna Schlosser, Andrea Allersdorfer, Gabriele Matschiner, Christine Rothe, Ulrich Moebius, Holbrook E Kohrt, Shane A Olwill

**Affiliations:** 1 Pieris Pharmaceuticals, Inc., Freising, Germany; 2 Stanford University, Stanford, CA, USA

## Background

CD137 is a potent costimulatory immunoreceptor and a member of the TNF-receptor (TNFR) superfamily. The receptor, also known as 4-1BB, is mainly expressed on activated CD4+ and CD8+ T cells, activated B cells, and natural killer (NK) cells. While multiple lines of evidence show that CD137 is a highly promising therapeutic target, current approaches are not designed to achieve a tumor-target driven activation. Thus, these approaches may display a limited therapeutic window due to peripheral T cell and NK cell activation, leading to unwanted toxicity. To overcome this limitation, we generated a bispecific protein therapeutic binding to CD137 and to a differentially expressed tumor target, HER2.

## Methods

Anticalin® proteins are 18 kD protein therapeutics derived from human lipocalins. We utilized phage display to generate an Anticalin protein binding to CD137 with high affinity and specificity. The CD137-specific Anticalin protein was genetically fused to a trastuzumab variant at different positions, yielding four different constructs covering a range of distances between the binding sites of the T cell-target and the tumor cell target. To minimize Fcγ-receptor interaction of the resulting bispecific and concomitant potential toxicity towards CD137-positive cells, the backbone of trastuzumab was switched from IgG1 to an engineered IgG4 isotype.

## Results

All four bispecific constructs bound the targets CD137 and HER2 with a nearly identical affinity compared to the parental building blocks, and both targets could be simultaneously bound. Compared to non-engineered trastuzumab, binding to human receptors FcγRI and FcγRIII was significantly reduced, while binding to the neonatal Fc receptor (FcRn) was retained. All constructs were found to be fully stable in human and mouse plasma, and in mice displayed pharmacokinetics similar to trastuzumab. HER2-dependent agonistic engagement of CD137 was demonstrated in ex-vivo T cell activation assays utilizing HER2-positive human cell lines. The functional activity of the bispecific constructs was found to be dependent on their geometry.

## Conclusion

We report the first bispecific therapeutic protein that targets the potent costimulatory immunoreceptor CD137 in a tumor-target dependent manner, utilizing HER2 as the tumor target. Compared to currently existing CD137-targeting antibodies, this approach has the potential to provide a more localized activation of the immune system with reduced peripheral toxicity. Bispecific T cell engagers based on CD137 and HER2 may have utility in HER2-positive cancers where there is a significant unmet medical need.

